# Solvent-Dependent Fluorescence Properties of CH_2_-*bis*(BODIPY)s

**DOI:** 10.3390/ijms232214402

**Published:** 2022-11-19

**Authors:** Alexander Kalyagin, Lubov Antina, Alexander Ksenofontov, Elena Antina, Mikhail Berezin

**Affiliations:** G.A. Krestov Institute of Solution Chemistry of the Russian Academy of Sciences, 1 Akademicheskaya Str., 153045 Ivanovo, Russia

**Keywords:** CH_2_-*bis*(BODIPY), fluorescent sensor, supramolecular structures, computational modelling

## Abstract

Biocompatible luminophores based on organic dyes, which have fluorescence characteristics that are highly sensitive to the properties of the solvating medium, are of particular interest as highly sensitive, selective, and easy-to-use analytical agents. We found that BODIPY dimers (2,2′-, 2,3′-3,3′-CH_2_-*bis*(BODIPY) (**1**–**3**)) demonstrate fluorescence characteristics with a high sensitivity to the presence of polar solvents. The intense fluorescence of **1**–**3** in nonpolar/low-polarity solvents is dramatically quenched in polar media (acetone, DMF, and DMSO). It has been established that the main reason for CH_2_-*bis*(BODIPY) fluorescence quenching is the specific solvation of dyes by electron-donating molecules (Solv) with the formation of stable supramolecular CH_2_-*bis*(BODIPY)·2Solv structures. Using steady-state absorption and fluorescence spectroscopy, time-resolved fluorescence spectroscopy, and computational modeling, the formation mechanism, composition, and structure of CH_2_-*bis*(BODIPY)·2Solv supramolecular complexes have been substantiated, and their stability has been evaluated. The results show the promise of developing fluorescent probes based on CH_2_-*bis*(BODIPY)s for detecting toxic N/O-containing compounds in solutions.

## 1. Introduction

Fluorescence spectroscopy is an attractive method for detecting specific analytes, including those in biological objects: the non-invasive early diagnosis of cancer, dysplastic lesions, and a number of other diseases [1,2,3,4,5]. In particular, regular contact with toxic N- and O-containing toxic compounds inevitably causes the disruption of important biological processes in the body, which must be detected early. Therefore, the study of compounds with fluorescence sensitivity to polar N- and O-electron-donor analytes is an urgent task. BODIPYs are among the successful candidates as fluorescent probes for analytes various types, such as toxic compounds, the polarity and nature of the medium, viscosity, etc. [6,7,8,9,10]. BODIPY derivatives, including oligomers with two or more BODIPY domains, have gained great interest due to their excellent optical properties, which can be purposefully controlled by modifying the chromophore molecular structure [11,12,13,14]. Thus, BODIPY dyes have many potential applications, both as components of optical devices and in bioimaging, photodynamic therapy, and sensorics [8,14,15,16,17,18,19,20].

**Scheme 1:**
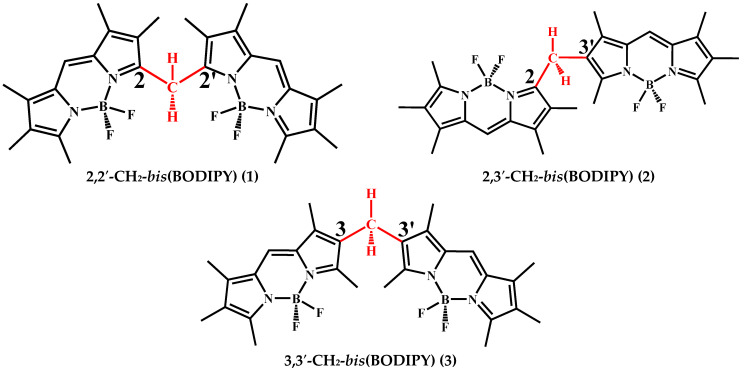
Chemical structures of BODIPY dimers (2,2′-, 2,3′-, 3,3′-CH_2_-*bis*(BODIPY) (**1**–**3**)).

Previous studies by our group [18] have focused on BODIPY dimers with a methylene spacer between the BODIPY domains. We have shown that the exciton splitting of the intense band in the electronic absorption spectra is typical not only for *bis*(BODIPY)s with a direct covalent bond between BODIPY domains [19,20,21,22,23,24,25,26,27,28,29,30,31] but also for CH_2_-*bis*(BODIPY) **1**–**3** dyes (Scheme 1) with chromophoric domains linked by a methylene group. In contrast to the moderately insensitive fluorescence characteristics of BODIPY monomeric dyes, the fluorescence quantum yield (Փ*_f_*) of dimeric CH_2_-*bis*(BODIPY) luminophores decreases by one or two orders of magnitude in the presence of polar proton- or electron-donating compounds in solutions. The fluorescence quantum yield of alkyl-substituted CH_2_-*bis*(BODIPY)s **1**–**3** decreases from 0.99 in nonpolar cyclic and linear hydrocarbons to 0.06 in polar proton-donor solvents and even to 0.008 in electron-donor organic solvents [21]. Thus, the sensitivity of CH_2_-*bis*(BODIPY)s **1**–**3**’s fluorescence characteristics to the environment’s nature is not inferior to the previously studied zinc(II) *bis*(dipyrromethate)s [22,23,24] and dimers with a direct covalent bond between the BODIPY domains [25,26,27,28].

Previously, we showed [29] that in alcohols, the increase in the intensity of non-radiative energy losses by molecules **1**–**3** in the excited state is caused by the formation of CH_2_-*bis*(BODIPY)·2alcohol supramolecular structures. The slight sensitivity of **1**–**3**’s fluorescence to viscosity is observed in highly viscous polar protic media. In the present work, a comprehensive analysis of fluorescence characteristics of **1**–**3** dimers with high sensitivity to the presence of electron-donating compounds was carried out using steady-state absorption and fluorescence spectroscopy, time-resolved fluorescence spectroscopy, and computational modeling.

## 2. Results and Discussion

### 2.1. Effect of the Nature of Polar Electron-Donating Solvents on CH_2_-bis(BODIPY)s ***1**–**3*** Spectral Properties

**Figure 1:**
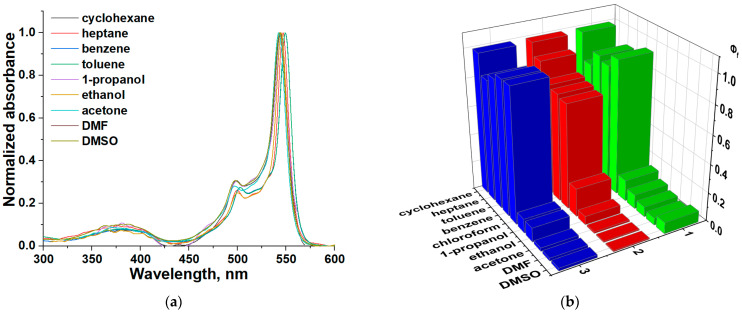
Normalized electronic absorption spectra of **3** in different solvents (**a**). The fluorescence quantum yield of **1**–**3** in nonpolar and polar solvents (**b**).

CH_2_-*bis*(BODIPY)s are characterized by intense absorption band splitting due to the intramolecular exciton interaction of non-conjugated BODIPY moieties [21] (Figure 1a and Table S1). The solvent nature does not significantly affect the absorption and emission bands position of compounds **1**–**3**. The most sensitive spectral characteristic of **1**–**3** to the medium’s nature was the fluorescence quantum yield (Figure 1b). The values of **1**–**3**’s fluorescence quantum yield range from 0.008 to 0.99 in the studied media (Table S1). The highest Փ*_f_* values of **1**–**3** dyes are observed in saturated and aromatic hydrocarbons (cyclohexane, heptane, benzene, and toluene) and chloroform [21]. This is due to the low polarity (polarizability) of these solvents, which solvate solutes only through universal interactions, in contrast to proton-donor (alcohols) and electron-donor solvents.

For comparison, the spectra and fluorescence quantum yield of **1**–**3** are given in nonpolar and polar proton-donating and electron-donating solvents (Figure 1a,b and Table S1). For the three structural isomers of CH_2_-*bis*(BODIPY), the same fluorescence-quenching trend is observed in alcohols [29], acetone, DMF, and DMSO. However, the fluorescence-quenching efficiency depends significantly on the polar solvent nature. Compared to cyclohexane, the Փ*_f_* of **1**–**3** decreases by 17 times in alcohols. However, maximum fluorescence quenching (up to 124 times) is observed in electron-donating media.

It is known [30] that the solvating environment’s effect on the change in the electronic levels of a molecule can be realized through two types of mechanisms. The first is based on macro-effects due to the macro-properties of solvents: the refractive index of the medium (*n*), dielectric constant (*ε*), viscosity (*η*), etc. The second type of dye fluorescence quenching is caused by specific interactions in the nearby solvate environment (donor–acceptor, hydrogen bonds, and π-stacking). We paid attention to the most informative electron-donating solvent parameters: basicity, electron-donating ability, and polarity. For compounds **1**–**3**, the correlation dependences of Փ*_f_* on various characteristics of solvents were plotted: Gutmann donor number (*DN*); Catalan’s solvent parameters: basicity (*SB*) and solvent polarity/polarizability (*SPP*); Kamlet–Taft solvent parameters: empirical parameter of solvent hydrogen-bond acceptor basicity (*β*) and dipolarity/polarizability parameters (π*); dielectric constant (*ε*); and dipole moment (*µ*) (Figure S1 and Table S2). Irrespective of the chosen scale, in all cases, the same trend of a decrease in the fluorescence quantum yield with an increase in the polarity, basicity, and electron-donor properties of the solvent was observed. The plots of Փ*_f_* vs. the solvent parameters (*DN*, *SB, SPP, β,* π*, ε, and *µ*) (Figure S1) deviate from linearity. Therefore, the high sensitivity of **1**–**3**’s luminescent characteristics to the solvent nature is due not only to the universal solvation of dye molecules by more polar and polarizable solvents but mainly to specific interactions between solvent molecules and the luminophores.

**Figure 2:**
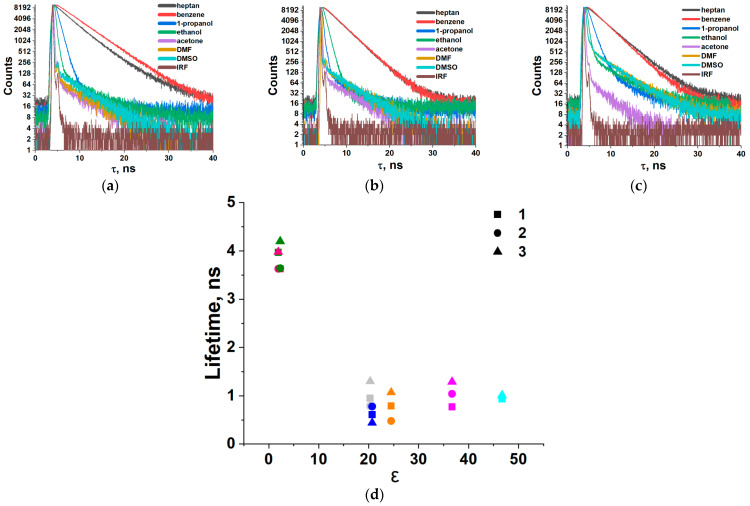
Time-resolved fluorescence decays of **1**–**3** in organic solvents (**a**–**c**) and the dependences of fluorescence lifetimes of **1**–**3** on organic solvent dielectric constant (**d**). ●—heptane; ●—benzene; ●—1-propanol; ●—ethanol; ●—acetone; ●—DMF; ●—DMSO.

The results of time-resolved fluorescence spectroscopy confirm this assumption (Figure 2; Tables S1 and S3). In inert solvents (heptane or benzene), the decay curves are monoexponential. In electron-donor solvents (acetone, DMF, or DMSO (Figure 2a–c)), the decay curves are biexponential. This may indicate the presence of an equilibrium mixture of the initial CH_2_-*bis*(BODIPY) complex and its specifically solvated form, CH_2_-*bis*(BODIPY)·nSolv, with a low fluorescence lifetime in the solution. The supramolecular complex with a short fluorescence lifetime (from 0.023 to 0.15 ns, Table S3) makes the maximum contribution to the total fluorescence lifetime. This indicates a significant shift of the equilibrium towards stable solvate structures of CH_2_-*bis*(BODIPY)·nSolv.

In Table S1, the values of the total lifetime and the calculated [30] radiative and non-radiative constants are given. The results show that for all **1**–**3** dyes in nonpolar media, the lifetime is noticeably longer (3.17–4.2 ns) than in acetone, DMF, and DMSO (0.44–1.30 ns). The value of the non-radiative constant is an order of magnitude higher in electron-donating solvents than in nonpolar media (Table S2). Obviously, in polar electron-donating solvents, non-radiative processes associated with rearrangements in the CH_2_-*bis*(BODIPY) solvation shell formed due to the active universal and specific interactions of the luminophores with the solvent molecules (Solv). The composition and stability of CH_2_-*bis*(BODIPY)·nSolv supramolecular complexes (where Solv is a polar electron-donating solvent molecule) are analyzed based on the results of experimental studies and computational modeling.

### 2.2. Investigation of Supramolecular Complexes of ***1**–**3*** with Electron-Donating Solvents (Acetone, DMF, and DMSO)

The influence of the composition of benzene–electron-donor solvent (acetone, DMF, or DMSO) binary mixtures on the spectral characteristics of **1**–**3** was studied experimentally. Complexes **1**, **2**, and **3** fluoresce intensely in benzene, with emission band maxima at 583, 569, and 559 nm, and the fluorescence quantum yields are 87, 76, and 94%, respectively (Table S1). With an increase in the polar component (Solv) molar fraction (χ), the intense band maxima in the absorption spectra of **1–3** shift to the blue region (up to 9 nm), along with a decrease in the absorption intensity at the first band maximum (Figure S2). This may indicate the stabilization of the system, which has a higher dipole moment in the excited state compared to the ground state. An increase in the electron-donor concentration in mixtures with benzene is accompanied by a blue shift (up to 15 nm) of the emission band maxima of **1**–**3** (Figure S3). The effect of the fluorescence quenching of **1**–**3** is observed even with small (χ = 0.2–0.6) additions of the electron-donor quencher in the binary solvent. The intensity and Ф*_f_* of **1**–**3** are close to the corresponding values in the electron donor at a higher mole fraction of the quencher in a binary solvent (Figures S2 and S3).

Complex **2** has fluorescence characteristics with the highest sensitivity to the presence of an electron donor, as in the case of alcohols (Figure S3) [29]. Compared to pure benzene in mixtures with a molar fraction of the electron donor χ = 0.3, the Ф*_f_* of **2** decreases by 6.7, 7.6, and 13.8 times in the presence of acetone, DMF, and DMSO, respectively (Figure S3b,e,h). The Ф*_f_* of compounds **1** and **3** in mixtures similarly decreases by 5.1, 5.9, and 8.4 and 4.7, 5.0, and 6.8 times respectively (Figure S3a,c,d,f,g,i). The first confirmation of the presence of specific interactions between **1**–**3** and acetone, DMF, and DMSO molecules was studied using the Stern–Volmer equation of fluorescence quenching (Equation (1)):

(1) $$I_{0}/I=1+K_{sv}\left[\mathrm{Slov}\right]$$

where *I* and *I*_0_ are the fluorescence intensities of **1**–**3** in the presence and absence of a quencher, respectively, *K*_SV_ is the fluorescence-quenching constant, and [*Solv*] is the quencher concentration.

**Figure 3:**
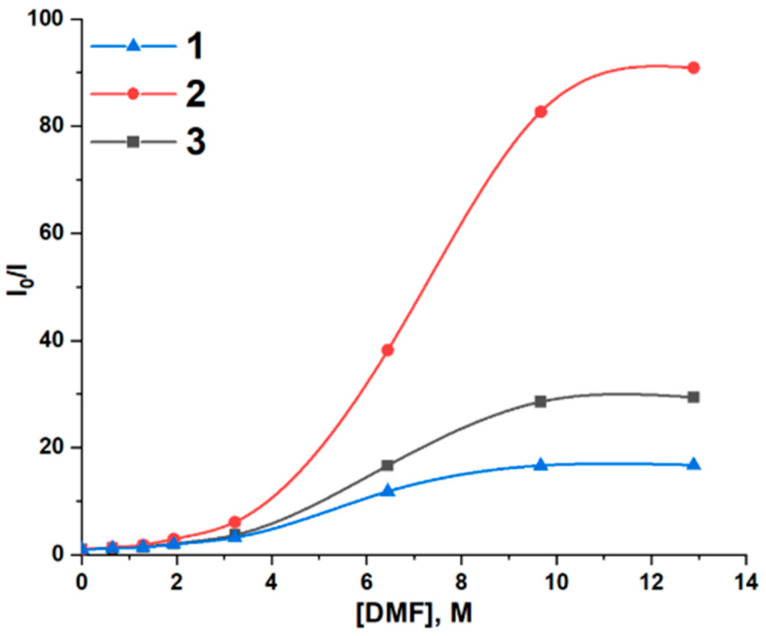
Stern–Volmer curves for fluorescence quenching of **1**–**3** (c = 2.0·10^−6^ mol/L) with the addition of various DMF concentrations.

The linearity deviation of Stern–Volmer plots in benzene–electron-donor mixtures indicates that the fluorescence quenching of **1**–**3** is dynamic and static in nature (Figure 3 and Figure S4).

**Figure 4:**
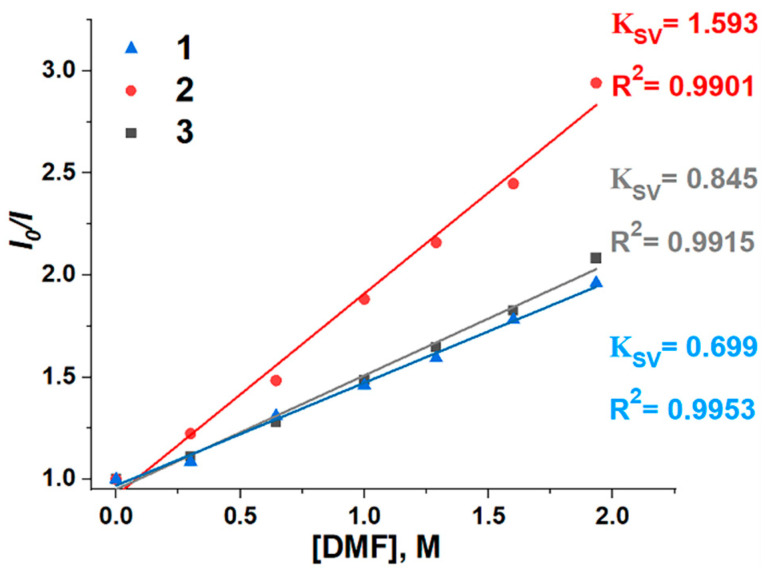
The linear fitting of the Stern–Volmer curve for the concentration range of DMF.

*K*_SV_ was estimated from the linear plot of *I*_0_/*I* vs. [Solv] up to ~2.6 M of the quencher (Figure 4 and Figure S5) in benzene–electron-donor systems. The analysis of the data obtained (Table 1; Figure 4 and Figure S5) showed that *K_SV_* values for **1**–**3** increase with an increase in the electron-donating ability in the series: acetone → DMF → DMSO.

The stability and composition of supramolecular structures of CH_2_-*bis*(BODIPY)s **1**–**3** were estimated from the results of UV/vis titration of **1**–**3** solutions in benzene–Solv (Solv: acetone, DMF, or DMSO). The stoichiometric composition of supramolecular complexes was determined from the slope of the graphical dependence lg[(*I_q_* – *I*_0_)/(*I*_∞_ − *I_q_*)] OT lg[Solv]) (Equation (2)) [31].

(2) $$K=\frac{\left(I_{q}-I_{0}\right)/\left(I_{\infty }-I_{0}\right)}{1-\left(I_{q}-I_{0}\right)/\left(I_{\infty }-I_{0}\right)}\cdot \frac{1}{{\left(c_{q}^{0}-c_{\left[bis\left(\mathrm{BODIPY}\right)\right]}^{0}\left(\frac{I_{q}-I_{0}}{I_{\infty }-I_{0}}\right)\right)}^{n}}$$

where $c_{q}^{0}$ the initial concentration of the electron donor (Solv); $c_{\left[bis\left(\mathrm{BODIPY}\right)\right]}^{0}$ is the initial concentration of CH_2_-*bis*(BODIPY)s **1–3** in benzene; *I*_0_ and *I* are **1–3**’s fluorescence intensities in the absence and presence of the electron donor (Solv), respectively; *I*_∞_ is **1–3**’s fluorescence intensity in a pure Solv quencher, and *n* is the stoichiometric coefficient.

The data obtained (Figure S6) indicate that **1**–**3** form supramolecular structures with the electron-donating molecules with the stoichiometric composition CH_2_-*bis*(BODIPY)·2Solv, which is consistent with the results of quantum chemical calculations. To estimate the thermodynamic constants of supramolecular complexation processes, we used the modified Benesi–Hildebrand equation (Equation (3)), which is traditionally used to determine the thermodynamic constants of processes from spectrofluorimetric titration data:

(3) $$\left(\;I_{\infty }-I_{0}\right)/(\;I_{x}-I_{0})=1+\left(1/K\right)(1/{\left[X\right]}^{n})$$

where *I*_0_ and *I_x_* are the fluorescence intensities of **1–3** in benzene in the absence and presence of Solv, respectively, *I*_∞_ is the fluorescence intensity of **1–3** in a pure quencher, *K_b_* is the binding constant, [*X*] is the quencher concentration, and n is the stoichiometric coefficient.

**Figure 5:**
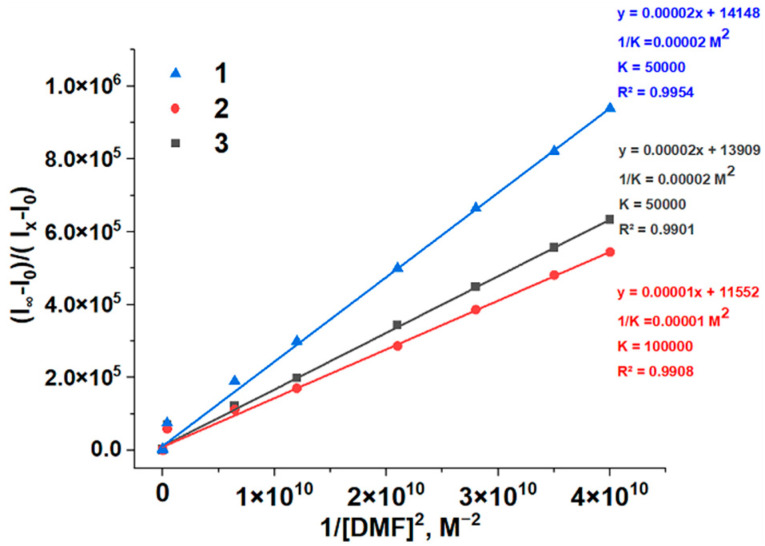
The Benesi–Hildebrand plots for determining the constants of additional coordination processes of **1**–**3** with DMF.

The lg*K* values of CH_2_-*bis*(BODIPY)·2Solv range from 2.7 to 5.4 (Figure 5 and Figure S7; Table 1). The results obtained indicate the relatively high stability of CH_2_-*bis*(BODIPY)·2Solv supramolecular structures with electron-donating analytes. The *lgK* values increase in the series **1** < **3** < **2**. (Table 1). This tendency is consistent with the regularity of the increase in the fluorescence sensitivity in the same series of **1**–**3**. Based on the results of our research, which have already been published in earlier papers with supramolecular structures of **1**–**3** with alcohols [29], we analyzed the effect of the solvents’ electron-donating ability on the fluorescence-quenching efficiency and the supramolecular structures’ stability (Figure S8).

The quenching constants and the stability of CH_2_-*bis*(BODIPY)·2Solv supramolecular structures increase with the increase in the electron-donating power of the solvent, and their analysis is included in the text of the article (marked in yellow). The results obtained are consistent with the conclusion that the main contribution to fluorescence quenching is made by specific interactions of the dye with the molecules of electron-donor solvents. Obviously, the differences in the stability of the molecular complexes of CH_2_-*bis*(BODIPY)·2Solv should be determined by the features of intermolecular and intramolecular interactions, which are discussed below based on the results of computational modeling.

### 2.3. DFT Calculations

**Figure 6:**
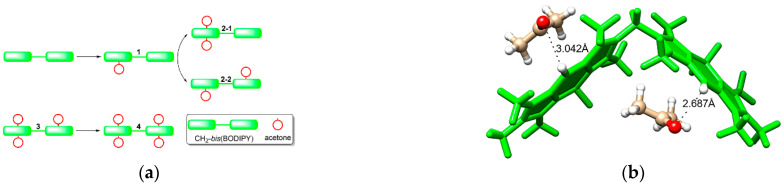
Schematic representation of the CH_2_-*bis*(BODIPY)·2acetone formation mechanism (**a**) and optimized structure of 3,3′-CH_2_-*bis*(BODIPY)·2acetone (**b**).

In Ref. [29], we demonstrated that CH_2_-*bis*(BODIPY) fluorescence quenching in proton-donor solvents is due to photoinduced electron (charge) transfer in the composition of CH_2_-*bis*(BODIPY)—nSolv supramolecular systems. In this study, using a similar DFT calculation algorithm and the example of CH_2_-*bis*(BODIPY)s with acetone systems, we estimated the formation probability, composition, and structural features of CH_2_-*bis*(BODIPY) supramolecular complexes with electron-donating molecules. Figure 6a schematically shows the CH_2_-*bis*(BODIPY)·(acetone)_n_ (n = 1–4) systems’ step-by-step formation mechanism, on the basis of which we determined their free energies of formation. An analysis of the free energy profiles shows that the most energetically favorable mechanism for CH_2_-*bis*(BODIPY)s is the sequential addition of acetone molecules to CH_2_-*bis*(BODIPY)s to form CH_2_-*bis*(BODIPY)·2acetone supramolecular systems (Figure S9). Supramolecular complexes of CH_2_-*bis*(BODIPY)·2acetone are formed through successive steps 1 and 2-2 by means of intermolecular contacts between the hydrogen atom of the methine spacer (H_ms_) of each BODIPY moiety and the acetone oxygen atom (O_solv_) (Figure 6b and Figure S10). The H_ms_∙∙∙O_solv_ bond lengths are 2.687–3.108 Å (Figure 6 and Figure S10; Table S4). The additional stabilization of these supramolecular systems is due to the presence of multipoint intermolecular contacts between the methyl moiety hydrogen atoms of the acetone molecule and one of the fluorine atoms of each BODIPY moiety. The free energy values confirm the thermodynamic stability of CH_2_-*bis*(BODIPY)·2acetone supramolecular complexes (Figure S9).

The results of TDDFT analysis made it possible to establish the effect of coordinated acetone molecules on the spectral properties of CH_2_-*bis*(BODIPY)s. The TDDFT spectra of CH_2_-*bis*(BODIPY)s are interpreted in detail in Ref. [21]. As in the case of the initial CH_2_-*bis*(BODIPY)s, in the TDDFT spectra of CH_2_-*bis*(BODIPY)·2acetone, the two most intense bands are due to H (HOMO)–L (LUMO) and H–L+1 electronic transitions (Figure S11 and Table S5). However, in the case of 3,3′-CH_2_-*bis*(BODIPY)·2acetone and 2,2′-CH_2_-*bis*(BODIPY)·2acetone, a new electronically excited state is found in the higher-energy region (5.41 and 5.32 eV, respectively). The formation of this state is associated with H-8–L+1 and H-9–L electronic transitions. In 3,3′-CH_2_-*bis*(BODIPY)·2acetone and 2,2′-CH_2_-*bis*(BODIPY)·2acetone, the transfer occurs between donor and acceptor moieties located at a closer distance. Due to the lower symmetry of 2,3′-CH_2_-*bis*(BODIPY), 2,3′-CH_2_-*bis*(BODIPY)·2acetone is characterized by the formation of two new electronically excited states (H-8–L and H-9–L+1; 5.31 and 5.41 eV). In new electronically excited states, an electron density transfer from acetone as the electron donor moiety to CH_2_-*bis*(BODIPY)·2acetone as the acceptor moiety is observed. It can be assumed that new low-lying electronically excited states are the result of ICT. Summarizing the results of the DFT study, we note that the fluorescence quenching of CH_2_-*bis*(BODIPY)s in acetone’s presence is associated with CH_2_-*bis*(BODIPY)·2acetone formation, which increases the efficiency of non-radiative conversion and the implementation of ICT.

## 3. Materials and Methods

The compounds *bis*(1,2,3,7,8-pentamethyl-2,2′-dipyrrolylmeten-9-yl)methane *bis*(difluoroborate) (**1**), (1,2,3,7,9-pentamethyl-2,2’-dipyrrolylmeten-8-yl)-(1,2,3,7,8-pentamethyl-2,2’-dipyrrolylmeten-9-yl)methane *bis*(difluoroborate) (**2**), and *bis*(1,2,3,7,9-pentamethyl-2,2′-dipyrrolylmeten-8-yl)methane *bis*(difluoroborate) (**3**) were synthesized according to proven technology [21]. The synthesis techniques and the results of the identification of **1–3** are presented in SI. Spectrophotometric-grade benzene, heptane, cyclohexane, toluene, ethanol, 1-propanol, DMF, DMSO, and acetone (for analysis, Panreac, Barcelona) were used without further purification. Solvent parameters (*DN*, *SB*, and *ε*) were taken from [32] and are listed in Table S2.

### 3.1. Spectroscopic Experiments

Electronic absorption and fluorescence spectra of **1**–**3** solutions were recorded on a CM 2203 spectrometer (SOLAR) in the range of molar concentrations from 10^−7^ to 10^−5^ mol/L, and the thickness of the absorbing layer was 10 mm at *T* = 25 ± 0.1 ° C. Fluorescence spectra were obtained at an optical density not higher than 0.1 at the excitation wavelength. The fluorescence quantum yields (Փ*_f_*) of **1**–**3** were obtained [30] with Equation (4):

(4) $${Փ}_{f}{}_{x}={Փ}_{st}\left(A_{st}/A_{x}\right)\left(S_{x}/S_{st}\right){\left(n_{x}/n_{st}\right)}^{2}$$

where Փ*_f_* is the fluorescence quantum yield of the substance (BODIPY), Փ*_st_* is the fluorescence quantum yield of the standard (Rhodamine 6G, Փ*_f_* = 94% [33]), *S* is integrated fluorescence intensity (area under spectrum), *A* is absorbance at the excitation wavelength (λ_ex_ = 520–525 nm for **1**–**3**), and *n* is the refractive index.

Time-resolved fluorescence measurements were carried out by means of a high-performance fluorescence lifetime and steady-state spectrometer FluoTime 300 (PicoQuant, Germany) with an LDH-P-C-500 laser (PicoQuant, Germany) as an excitation source. The instrument response function (IRF) of the system was measured with the stray light signal of a dilute colloidal silica suspension (LUDOX^®^). The fluorescence decay curves were measured at the maxima of the emission peaks, and the fluorescence lifetimes were obtained by the reconvolution of the decay curves using the EasyTau 2 software package (PicoQuant, Germany). For nonpolar solvents (cyclohexane and benzene), the monoexponential decay model was applied. For polar electron-donating solvents (acetone, DMF, and DMSO), the biexponential fluorescence decay model was applied.

### 3.2. Computational Modeling

All calculations were carried out using Gaussian 16 [34]. To analyze the mechanism of CH_2_-*bis*(BODIPY)–acetone system formation, geometry optimization and harmonic frequency calculations were performed with ωB97X-D/def2-TZVP [35,36]. The vertical electronic transitions were computed by the TDDFT method employing CAMB3LYP/def2-TZVP [37]. The SMD model [38] was applied to simulate the acetone medium. ChemCraft 1.8 (www.chemcraftprog.com, accessed on 30 October 2022) was used for analyses of results and molecular graphics.

## 4. Conclusions

The spectral properties and fluorescence quenching effect of CH_2_-*bis*(BODIPY)s in polar electron-donating solvents (acetone, DMF, and DMSO) were analyzed in comparison with nonpolar solvents. The fluorescence quantum yield of **1**–**3** decreases from 0.99 in nonpolar cyclic and linear hydrocarbons and weakly polar chloroform to 0.008 in electron-donor organic solvents. In polar solvents, there is a complication of the law of fluorescence lifetime decay. The results show that for all dyes in nonpolar and weakly polar solvents, **1**–**3**’s total lifetime is noticeably higher (3.17–4.2 ns) than in acetone, DMF, and DMSO (0.44–1.30 ns). The high fluorescence sensitivity of CH_2_-*bis*(BODIPY)s to the polarity, basicity, and electron-donating ability of solvents is due to CH_2_-*bis*(BODIPY)·2Solv supramolecular structure formation. The values of the fluorescence-quenching constants (*K*_SV_) of CH_2_-*bis*(BODIPY)s **1**–**3** increase with the growth of the solvent’s electron-donor properties: acetone → DMF → DMSO. The equilibrium constants (lg*K*) of the supramolecular complex formation processes, calculated using the modified Benesi–Hildebrand equation, are in the range from 2.7 to 5.4 and indicate the higher stability of CH_2_-*bis*(BODIPY) supramolecular structures with electron-donating solvents compared to alcohols. The results form the basis for the development of fluorescent probes based on CH_2_-*bis*(BODIPY)s for monitoring the complex parameter of the cellular structure polarity or the detection of electron-donating compounds (acetone, DMF, and DMSO) in solutions and biosystems.

## Figures and Tables

**Table 1:** The values of *K_SV_* and lg*K* for CH_2_-*bis*(BODIPY)·2Solv supramolecular structures.

	Luminophores		
	1	2	3
**Solv**	*K_SV_*, M^−1^; (lg*K*)		
Acetone	0.048 (2.7)	0.095 (4.2)	0.056 (3.5)
DMF	0.699 (4.7)	1.593 (5.0)	0.845 (4.8)
DMSO	1.913 (5.1)	4.211 (5.4)	2.590 (5.2)

Note: The error in determining lg*K* values from fluorometric titration data does not exceed 7–10%.

## Data Availability

The raw data supporting the conclusions of this article will be made available by the authors, without undue reservation.

## Supplementary Materials

The are available online at https://www.mdpi.com/article/10.3390/ijms232214402/s1. References [21,29,32,39] are cited in the supplementary materials.
