# Supplementary material for: Solvent-Dependent Fluorescence Properties of CH2-bis(BODIPY)s
Source: Int J Mol Sci. 2022 Nov 19;23(22):14402. doi: 10.3390/ijms232214402 (PMC9695564; doi:10.3390/ijms232214402)
Supplement: Supplementary file 1 [file ijms-23-14402-s001.zip › ijms-1999849-supplementary.pdf]

Supplementary Material

Solvent-Dependent Fluorescence Properties of CH_2-*bis*(BODIPY)s

Alexander Kalyagin, Lubov Antina, Alexander Ksenofontov, Elena Antina * and Mikhail Berezin

G.A. Krestov Institute of Solution Chemistry of the Russian Academy of Sciences, 1 Akademicheskaya Str., 153045 Ivanovo, Russia
* Correspondence: eva@isc-ras.ru

Synthetic procedures for 1-3

Compounds **1-3** were synthesized according to the procedure presented in [1]

Identification of synthesized ***bis*(1,2,3,7,8-pentamethyl-2,2′-dipyrrolylmeten-9-yl)methane *bis*(difluoroborate), (1):** FTIR, *v* cm^{-1}: 2963, 2918, 2859 (C-H), 1601, 1406 (C=N), 1469 (C=C), 1363 (B=N), 1192, 1142 (B-F). ^1H NMR ($CDCl_3$): 7.02 s (H, CH); 6.08 s (H, CH); 4.11 s (2H, CH_2); 2.52 s (3H, CH_3); 2.50 s (3H, CH_3); 2.43 s (3H, CH_3); 2.19 s (3H, CH_3); 2.16 s (3H, CH_3); 2.13 s (3H, CH_3); 2.09 s (3H, CH_3); 1.96 s (3H, CH_3); 1.95 s (3H, CH_3); 1.60 s (3H, CH_3); ^{11}B NMR ($BF_3{\cdot}OEt_2$, $CDCl_3$): 1.07 (t, *J* (^{11}B – ^{19}F)=31.48 Hz), 0.84 (s); Mass spectrum, m/z (I, %): 536.9 (85) $[M]^+$. Calcd, %: C, 64.96; H, 6.39; N, 10.45; Found: C, 64.87; H, 6.42; N, 10.53.

Identification of synthesized ***bis*(1,2,3,7,9-pentamethyl-2,2'-dipyrrolylmeten-8-yl)-(1,2,3,7,8-pentamethyl-2,2'-dipyrrolylmeten-9-yl)methane *bis*(difluoroborate)**, (**2**): FTIR, *v* cm^{-1}: 2965, 2920, 2859 (C-H), 1601, 1406 (C=N), 1470 (C=C), 1370 (B=N), 1188, 1134 (B-F); ^1H NMR ($CDCl_3$): 7.02 s (H, CH); 6.08 s (H, CH); 4.11 s (2H, CH_2); 2.52 s (3H, CH_3); 2.50 s (3H, CH_3); 2.43 s (3H, CH_3); 2.19 s (3H, CH_3); 2.16 s (3H, CH_3); 2.13 s (3H, CH_3); 2.09 s (3H, CH_3); 1.96 s (3H, CH_3) 1.95 s (3H, CH_3); 1.60 s (3H, CH3); ^{11}B NMR ($BF3{\cdot}OEt_2$, $CDCl_3$): 1.18 (q, *J* (^{11}B – ^{19}F)=32.70 Hz); Mass spectrum, m/z (I, %): 536.8 (100) $[M]^+$; Calcd, %: C, 64.96; H, 6.39; N, 10.45; Found: C, 64.87; H, 6.42; N, 10.53; Calcd, %: C, 64.96; H, 6.39; N, 10.45;Found: C, 64.85; H, 6.44; N, 10.41.

Identification of synthesized ***bis*(1,2,3,7,9-pentamethyl-2,2′-dipyrrolylmeten-8-yl)methane *bis*(difluoroborate), (3):** FTIR, *v* cm^{-1}: 2972, 2919, 2870 (C-H), 1601, 1404 (C=N), 1472 (C=C), 1374 (B=N), 1142, 1066 (B-F); ^1H NMR ($CDCl_3$): 7.08 s (2H, ms-CH); 3.60 s (2H, ms-CH_2); 2.63 s (6H, CH_3); 2.53 s (6H, CH_3); 2.29 s (6H, CH_3); 2.18 s (6H, CH_3); 2.02 s (6H, CH_3); ^{11}B NMR ($BF3{\cdot}OEt_2$, $CDCl_3$): 0.96 (t, *J* (^{11}B – ^{19}F)=33.33 Hz); Mass spectrum, m/z (I, %): 536.8 (60) $[M]^+$; Calcd,%: C, 64.96; H, 6.39; N, 10.45; Found: C, 64.91; H, 6.45; N, 10.48.

Table S1. Spectroscopic (absorption maxima, molar absorption coefficients, exciton splitting, emission maxima, Stokes shift) and photophysical parameters (fluorescence quantum yield, lifetime, radiative and non-radiative rate constants) of **1-3** in organic solvents, 298 K.

Solvent	λ_{abs}, nm (lgε)	$\Delta\nu_{es}$, cm^{-1}	λ_{fl}, nm	$\Delta\nu_{SS}$, cm^{-1}	Φ_f	$K_{rad}\cdot 10^{-8}$, s^{-1}	$K_{nrad}\cdot 10^{-8}$, s^{-1}	τ_{fl}, ns
**1**								
cyclohexane	564; 501; 385	2230	580	490	0.99	2.94	0.03	3.37
heptane	562; 500; 378	2207	578	493	0.80	2.22	0.30	3.97
benzene	566 (5.01); 500; 380	2332	583	515	0.87	2.40	0.36	3.63
toluene	566 (5.09); 501; 385	2292	585	574	0.90	2.83	0.31	3.18
chloroform	564 (5.11); 500;381	2270	581	519	0.95	2.75	0.15	3.46
1-propanol	558 (5.13); 497; 385	2200	577	590	0.15	1.58	8.95	0.95
ethanol	557 (5.08); 496; 377	2208	573	501	0.10	1.27	11.39	0.79
acetone	555; 496; 380	2143	571	505	0.07	1.15	1.52	0.61
DMF	556 (5.04); 495; 382	2216	583	833	0.05	0.65	12.3	0.77
DMSO	556; 496; 381	2176	581	774	0.08	0.85	9.79	0.94
**2**								
cyclohexane	551; 500; 384	1821	568	543	0.99	2.96	0.03	3.34
heptane	550; 499; 374	1858	567	545	0.9	2.48	0.28	3.63
benzene	553 (5.14); 502; 383	1837	569	508	0.76	2.09	0.66	3.64
toluene	553 (5.14); 502;377	1837	569	508	0.78	2.46	0.70	3.17
chloroform	552 (5.09); 501;384	1844	569	541	0.74	2.11	0.74	3.51
1-propanol	549 (5.18); 498; 378	1865	563	453	0.20	2.30	9.20	0.79
ethanol	548 (5.06); 497; 370	1873	554	197	0.06	1.25	19.58	0.48

acetone	546; 497; 383	1806	563	553	0.012	0.15	12.7	0.78
DMF	547 (5.06); 496; 379	1880	551	133	0.008	0.077	9.54	1.04
DMSO	548; 496; 381	1913	558	327	0.008	0.083	10.2	0.97
**3**								
cyclohexane	548; 502; 389	1672	554	197	0.99	2.96	0.03	-
heptane	547; 500; 377	1718	554	231	0.85	2.14	3.77	3.98
benzene	549 (5.15); 502;384	1672	559	326	0.94	2.24	0.14	4.2
toluene	549 (5.11); 502; 378	1672	558	294	0.90	2.49	0.28	3.61
chloroform	548 (5.09); 502; 380	1672	558	327	0.94	2.37	0.15	3.97
1-propanol	545 (5.10); 497; 379	1772	554	298	0.10	0.77	6.92	1.30
ethanol	544 (5.20); 497; 378	1739	551	233	0.10	0.94	8.41	1.07
acetone	543; 497; 371	1705	553	333	0.024	0.55	22.2	0.44
DMF	543 (5.08); 498; 380	1664	552	300	0.02	0.16	7.60	1.29
DMSO	544; 498; 378	1698	552	266	0.02	0.20	9.70	1.01

Table S2. Solvent properties and polarity parameters: Gutmann donor number (*DN*); Catalan's solvent parameter: basicity (*SB*), solvent polarity/polarizability (*SPP*); Kamlet-Taft solvent parameters: empirical parameter of solvent hydrogen-bond acceptor basicity (*β*), dipolarity/polarizability parameters (*π**); dielectric constant (*ε*), dipole moment (μ) [2,3].

Solvent	DN	SB	SPP	*β*	*π**	ε	μ
heptane	0	0.08	0.53	0	-0.08	1.92	0
benzene	0.1	0.12	0.67	0.1	0.59	2.28	0
chloroform	4,8	0.07	0.75	0.1	0.58	4.9	1.15
1-propanol	19.8	0.78	0.85	0.9	0.52	20.3	1.65
ethanol	19.6	0.66	0.85	0.75	0.54	24.5	1.68
acetone	17	0.48	0.88	0.43	0.71	20.7	2.84
DMF	26.6	0.61	0.95	0.69	0.88	36.7	3.93
DMSO	29.8	0.65	1	0.76	1	45.0	4.10

Table S3. The amplitudes (a_i) and lifetimes (τ_i) of individual components intensity-weighted mean lifetimes used to calculate the total lifetime of **1-3** in organic solvents.

	**1**		**2**		**3**	
	τ_i. ns	a_i	τ_i. ns	a_i	τ_i. ns	a_i
ethanol	0.25 ± 0.0018	28.25	0.20 ± 0.002	52.18	0.29 ± 0.004	37.99
	5.79 ± 0.17	0.1316	5.58 ± 0.092	0.1031	5.14 ± 0.066	0.4099
1-propanol	0.85 ± 0.004	18.55	0.79 ± 0.002	20.770	0.98 ± 0.005	18.233
	4.65 ± 0.32	0.0924	6.49 ± 0.85	0.0360	4.59 ± 0.15	0.372
acetone	0.12±0.0008	37.33	0.11±0.001	37.47	0.15±0.002	30.00
	5.58±0.14	0.077	5.85±0.069	0.093	3.77±0.061	0.1017
DMF	0.037±0.001	116.2	0.068+0.015	98	0.048±0.0066	234
	5.90±0.088	0.104	4.95+0.015	7.36	5.072±0.038	0.728
DMSO	0.027±0.002	182	0.0228±0.0023	232	0.029±0.005	483
	5.80±0.16	0.1572	5.58±0.14	0.194	4.75±0.039	0.772

Table S4. Hydrogen bond parameters for CH_2-*bis*(BODIPY)**1-3**·2acetone systems.

	CH_2-*bis*(BODIPY)**1**·2acetone	CH_2-*bis*(BODIPY)**2**·2acetone	CH_2-*bis*(BODIPY)**3**·2acetone
$O_{\text{solv}} \cdots H_{ms}$. Å	3.088	3.108	3.042
$O_{\text{solv}} \cdots H_{ms}$. Å	2.917	2.850	2.687
$\text{H}_{\text{solv}} \cdots F_1$ Å	2.609	2.432	2.420
$\text{H}_{\text{solv}} \cdots F_2$ Å	2.399	2.466	2.690
$\text{H}_{\text{solv}} \cdots F_3$ Å	2.464	2.532	2.546
$\text{H}_{\text{solv}} \cdots F_4$ Å	2.506	2.607	2.394

Table S5. The results of TDDFT analysis of CH_2-*bis*(BODIPY)**1-3**·2acetone.

**Excited State**	**Transition, (%)**	**Energy, eV (nm)**	**Oscillator strength**	**Frontier molecular orbitals**
		**CH_2-*bis*(BODIPY)3·2acetone**		
S_1	H – L+1, (16)	2.68 (463)	0.966	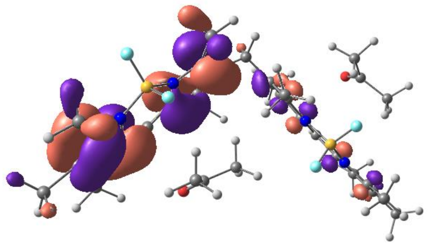 **H** 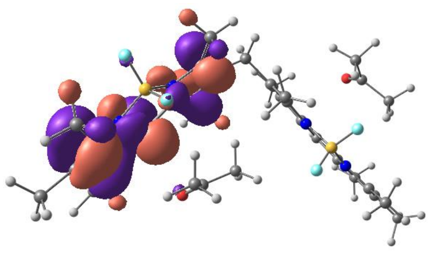 **L+1**

S_2	H – L, (21)	2.85 (436)	0.481	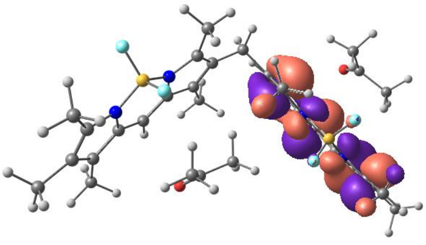<br>**L**
S_{16}	H-8 – L+1, (38)	5.41 (230)	0.131	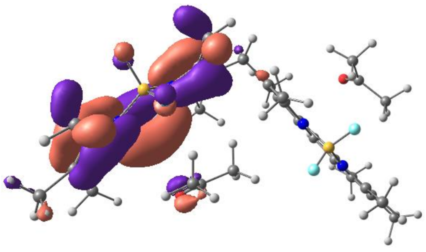<br>**H-8**
S_{26}	H-1 – L+5, (21)	6.15 (202)	0.125	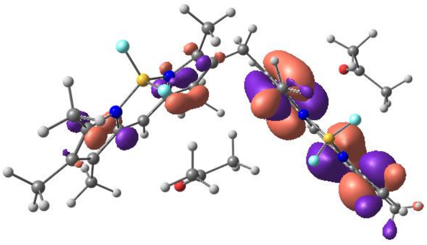<br>**H-1**<br>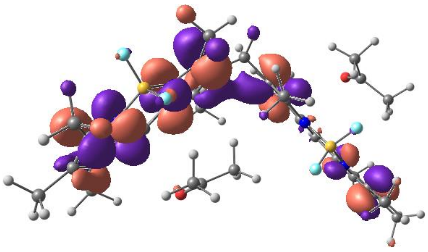<br>**L+5**
S_{29}	H-1 – L+6, (19)	6.45 (192)	0.225	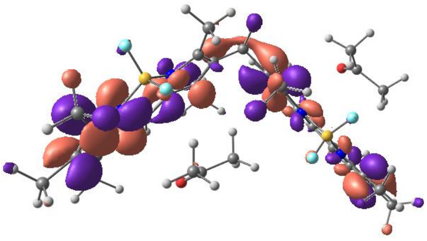<br>**L+6**
		**CH$_2$-*bis*(BODIPY)2·2acetone**		
S_1	H – L+1, (25)	2.67 (464)	1.312	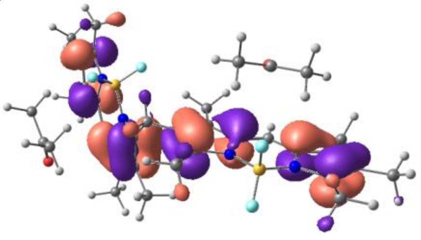<br>**H**

				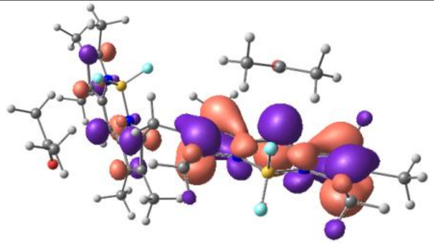 **L+1**
S_2	H – L, (14)	2.85 (435)	0.212	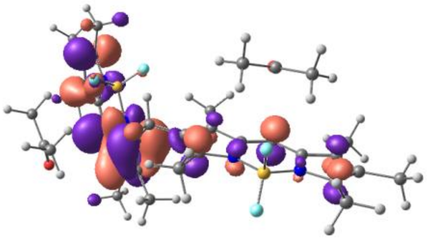 **L**
S_{15}	H-8 – L, (39)	5.31 (233)	0.120	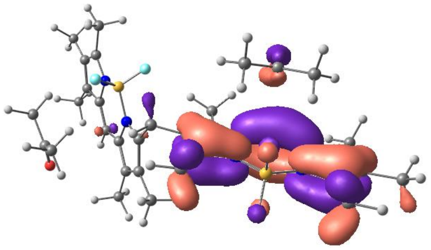 **H-8**
S_{16}	H-9 – L+1, (40)	5.41 (229)	0.128	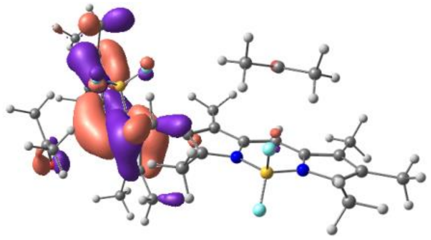 **H-9**
S_{25}	H – L+4, (29)	6.07 (204)	0.177	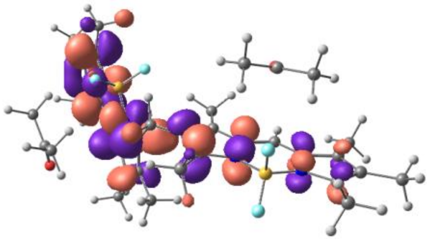 **L+4**
S_{30}	H-1 – L+7, (18)	6.62 (187)	0.156	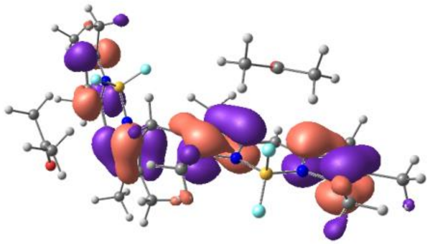 **H-1**

				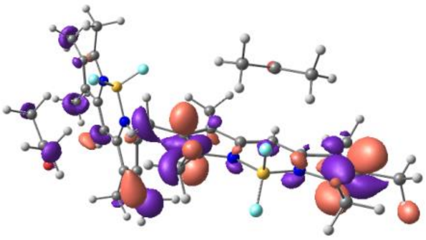 L+7
**CH_2-*bis*(BODIPY)1·2acetone**				
S_1	H – L+1, (16)	2.65 (468)	0.858	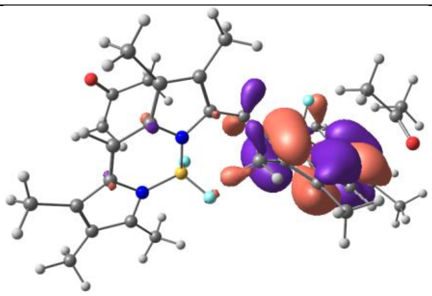 **H** 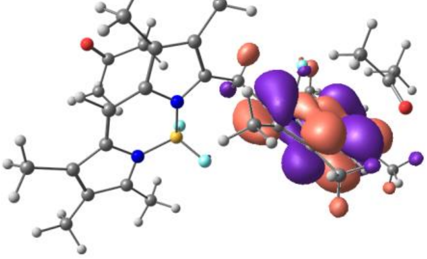 **L+1**
S_2	H-1 – L, (18)	2.86 (434)	0.548	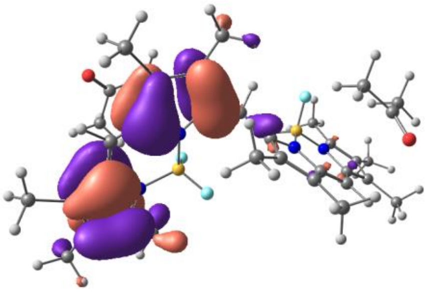 **H-1** 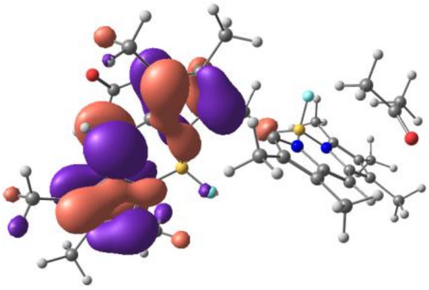 **L**
S_{15}	H-9 – L, (19)	5.32 (233)	0.201	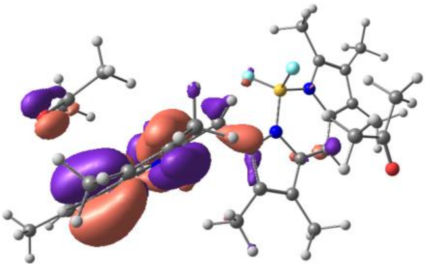

S_{25}	H – L+5, (28)	6.06 (205)	0.186
S_{29}	H-1 – L+7, (21)	6.53 (190)	0.168
S_{30}	H – L+7, (15)	6.56 (189)	0.114

H-9

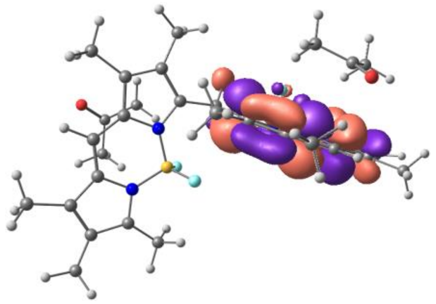

L+5

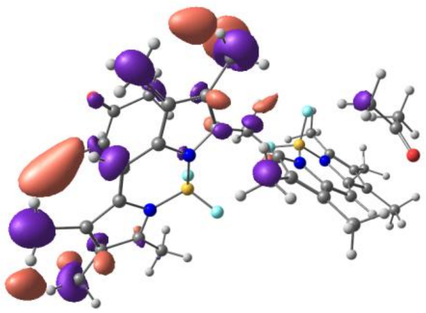

L+7

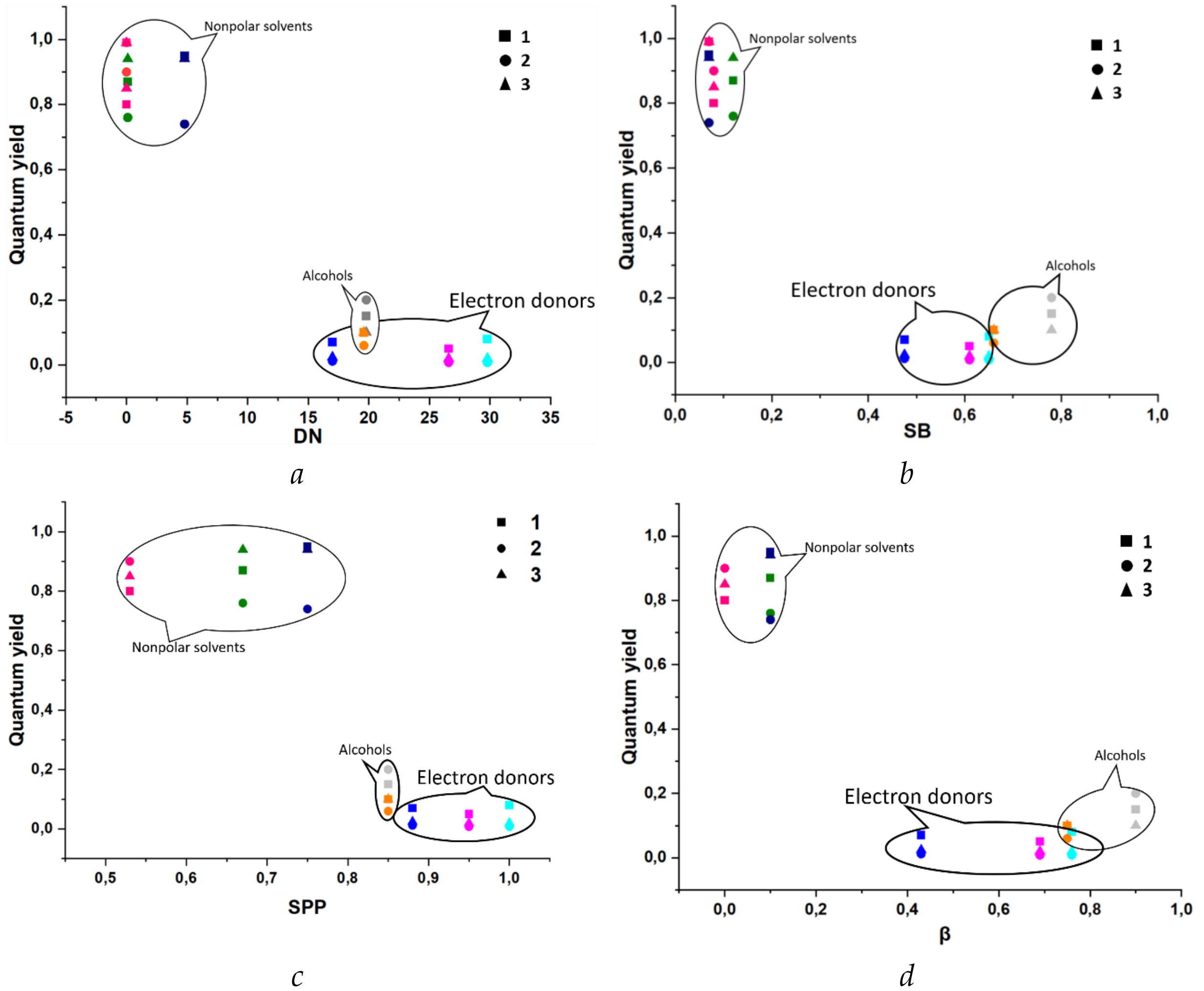

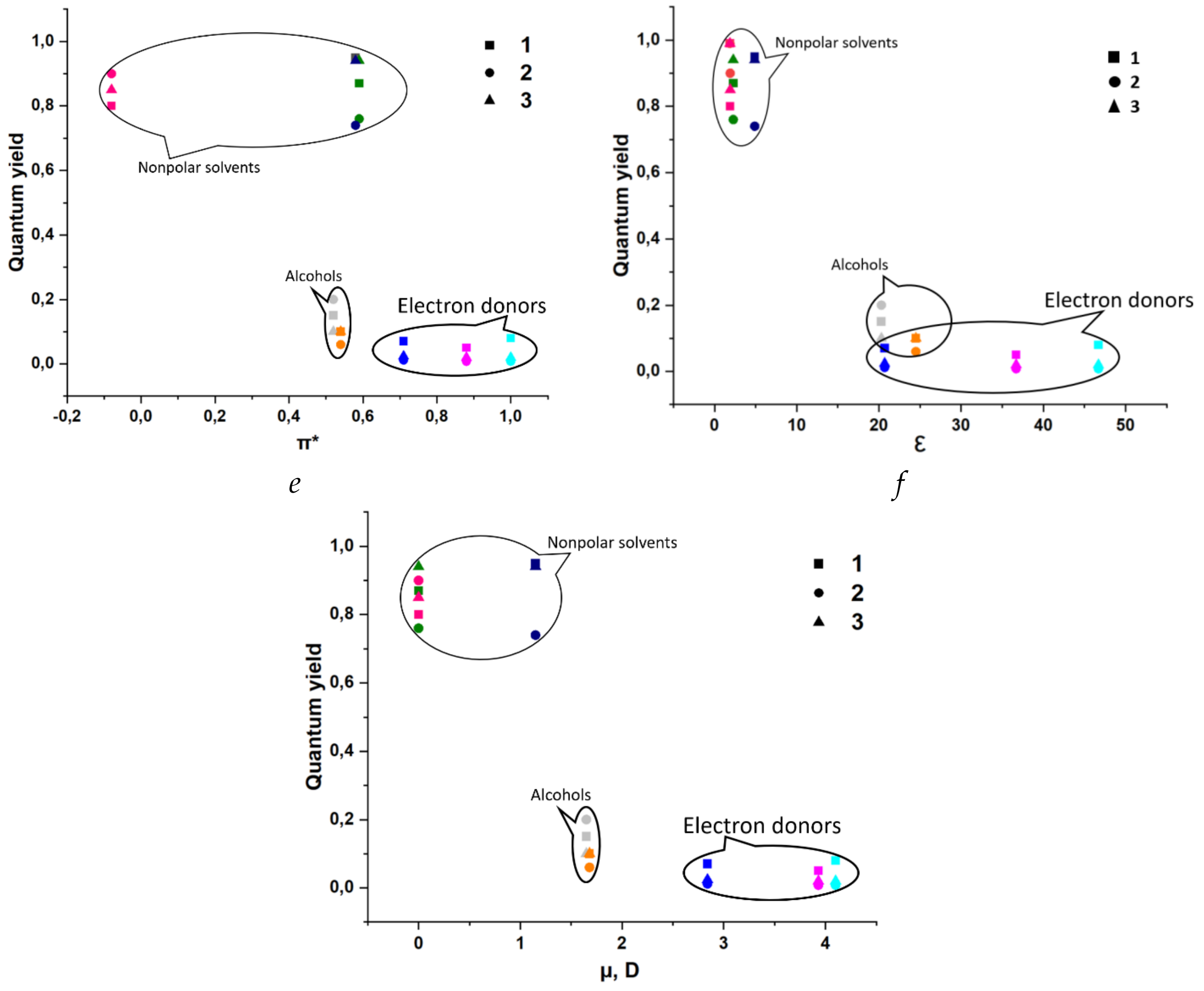

g

Figure S1. The dependences of the fluorescence quantum yield of **1-3** on the parameters of the solvents: Gutmann donor number (a); Catalan’s solvent parameter: basicity (b), solvent polarity/polarizability (c); Kamlet-Taft solvent parameters: empirical parameter of solvent hydrogen-bond acceptor basicity (d), dipolarity/polarizability parameters (e); dielectric constant (f), dipole moment (g).

● – heptane; ● – benzene; ● – chloroform; ● – 1-propanol; ● – ethanol; ● – acetone; ● – DMF; ● – DMSO

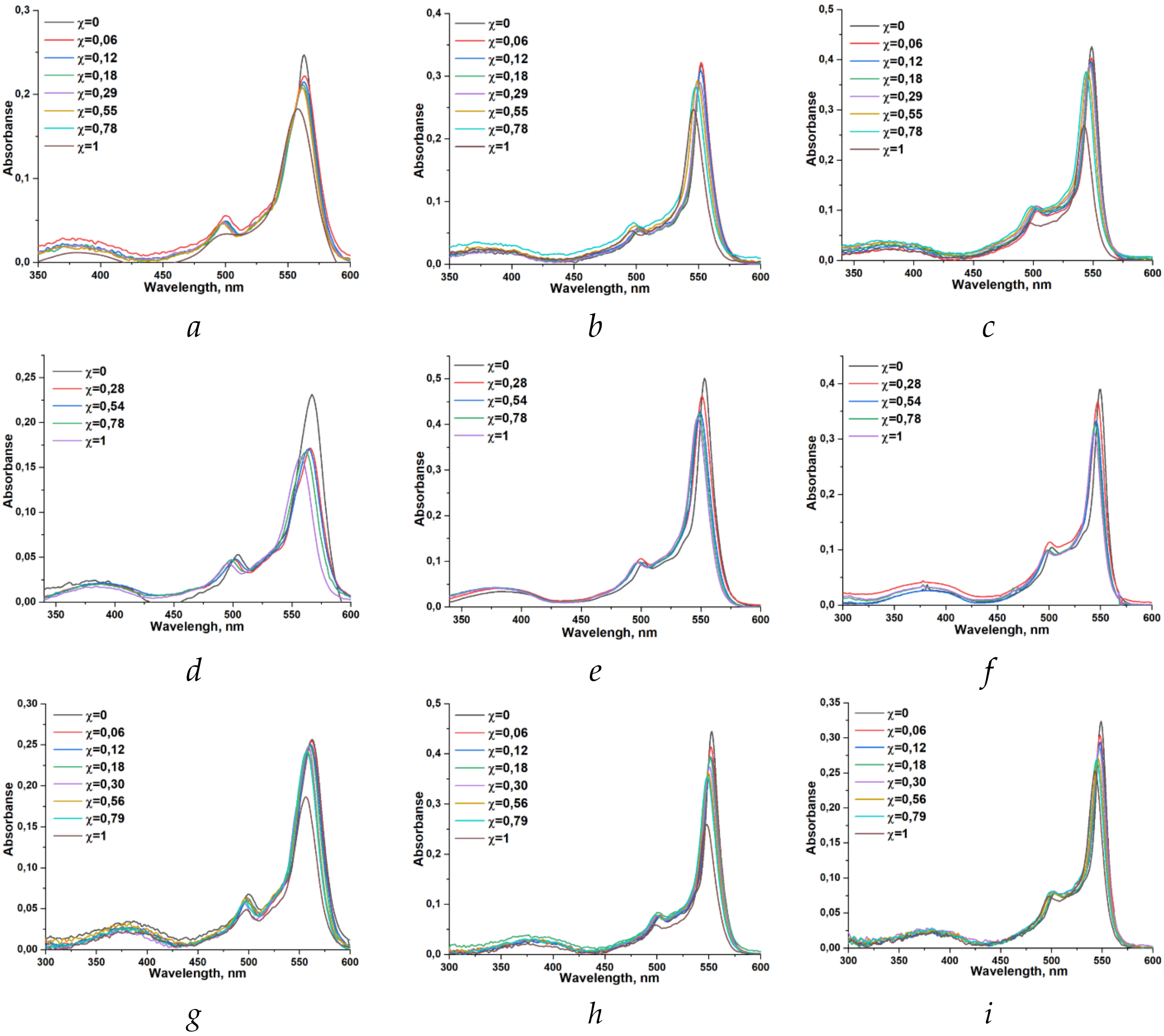

Figure S2. Absorption spectra of **1** (a, d, g), **2** (b, e, h), **3** (c, f, i) in benzene/electron donor mixtures with different molar fraction of acetone (a, b, c), DMF (d, e, f) and DMSO (g, h, i), $C_{bis(BODIPY)} \approx 2.0 \cdot 10^{-6}$ mol/l.

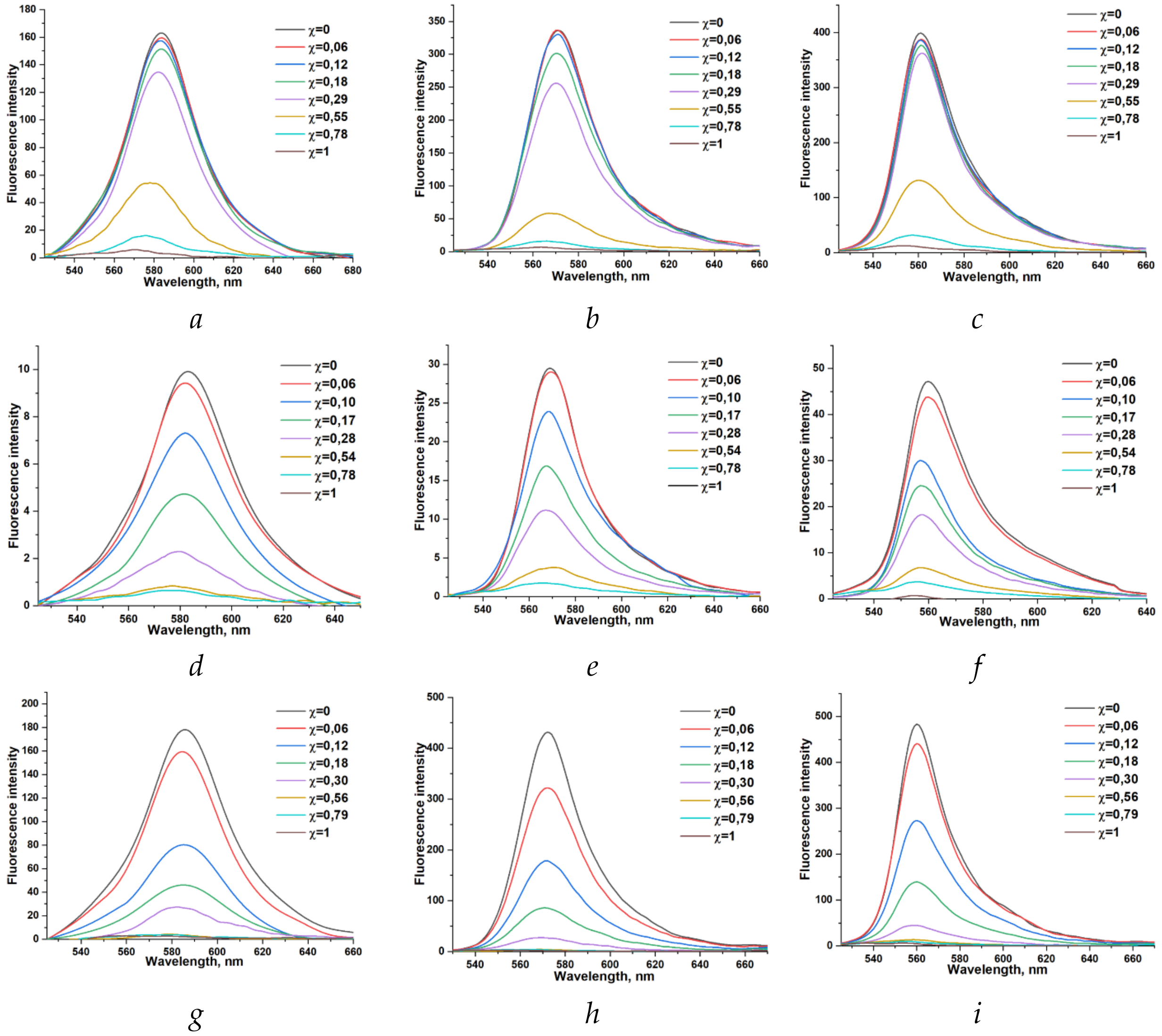

Figure S3. Emission spectra of **1** (a, d, g), **2** (b, e, h), **3** (c, f, i) in benzene/electron donor mixtures with different molar fraction of acetone (a, b, c), DMF (d, e, f) and DMSO (g, h, i), $C_{bis(BODIPY)} \approx 2.0 \cdot 10^{-6}$ mol/l.

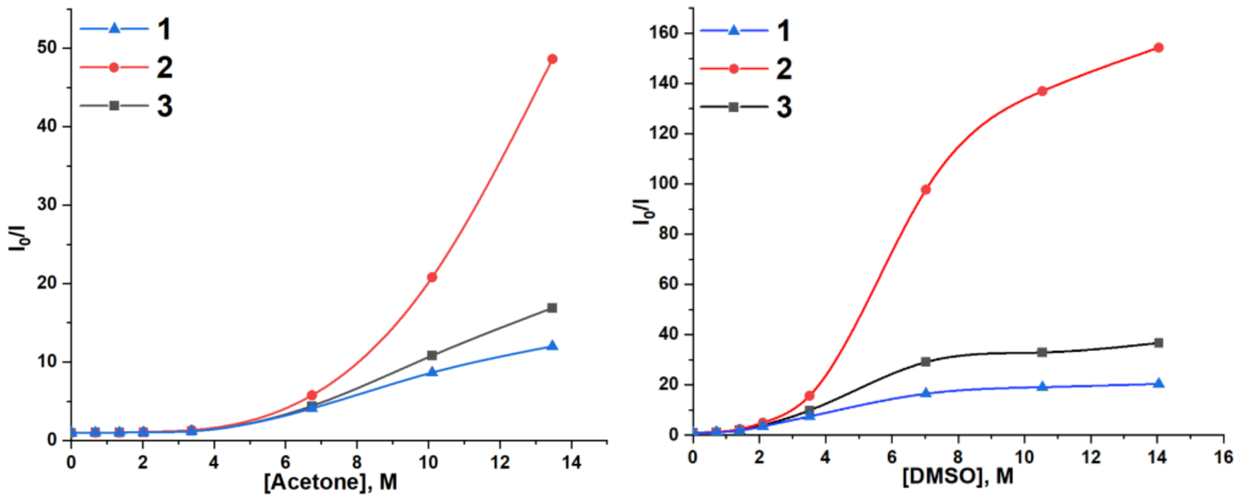

a *b*

Figure S4. Stern-Volmer curves for fluorescence quenching of **1**-**3** (c=2.0·10^{-6} mol/l) at the addition of acetone (a) and DMSO (b) various concentrations.

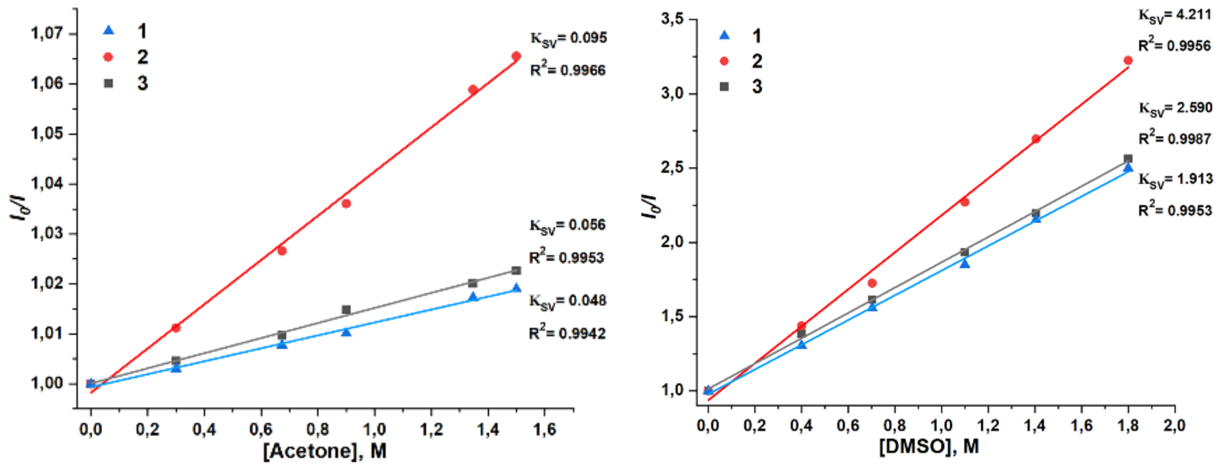

a *b*

Figure S5. The linear fitting of the Stern-Volmer curve for the concentration range of acetone (a) and DMSO (b).

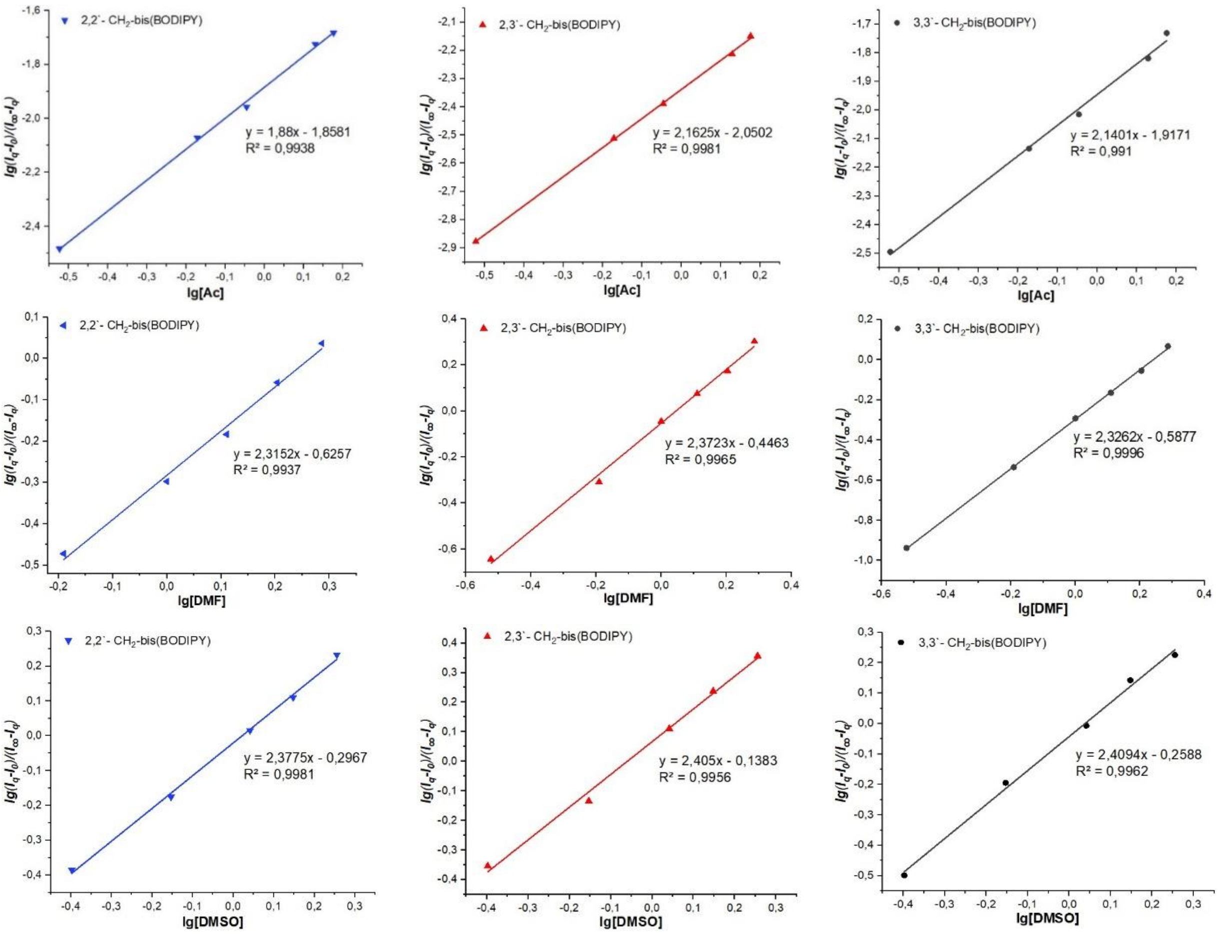

a *b* *c*

Figure S6. The dependences of lg[(I_q-I_0)/(I_∞-I_q)] of **1** (a), **2** (b), **3** (c) on the analyte (acetone, DMF, DMSO).

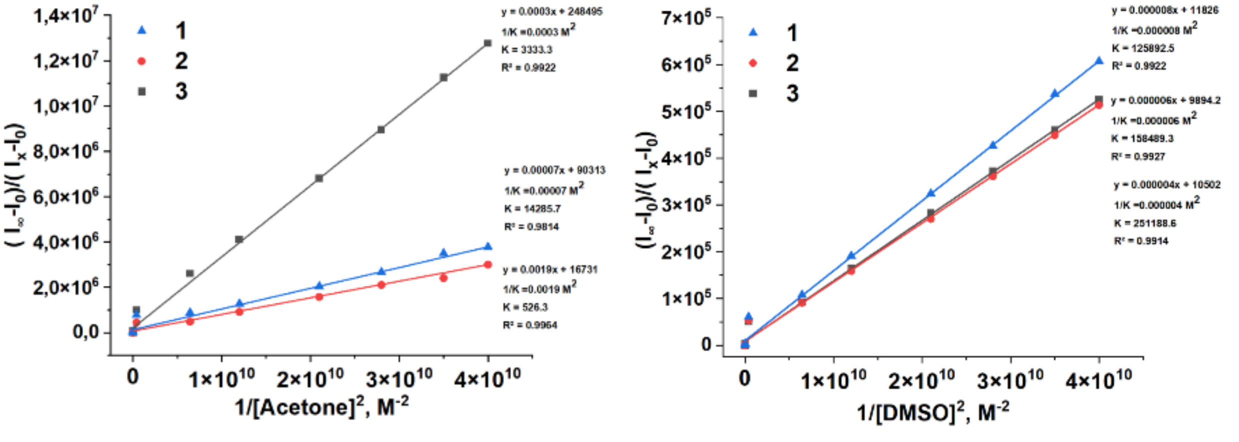

a *b*

Figure S7. The Benesi-Hildebrand plots for determining the constants of additional coordination processes **1**-**3** with acetone (a) and DMSO (b).

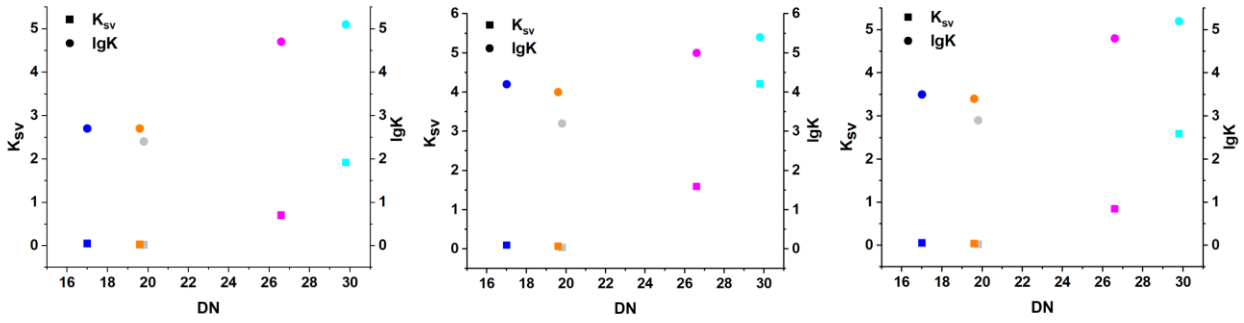

a *b* *c*

Figure S8. The dependencies of the values of *Ksv* and *lgK* of **1** (a), **2** (b), **3** (c) on the Gutmann donor number. ● – 1-propanol; ● – ethanol; ● – acetone; ● – DMF; ● – DMSO (values for alcohols are taken from [4]).

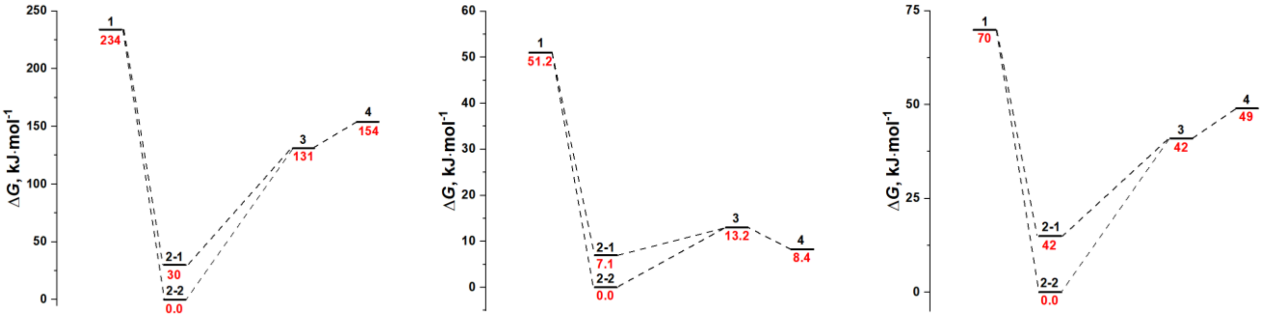

a *b* *c*

Figure S9. The relative free energy profiles for CH_2-*bis*(BODIPY)**3**·2acetone (*a*), CH_2-*bis*(BODIPY)**1**·2acetone (*b*), and CH_2-*bis*(BODIPY)**2**·2acetone (*c*) formation.

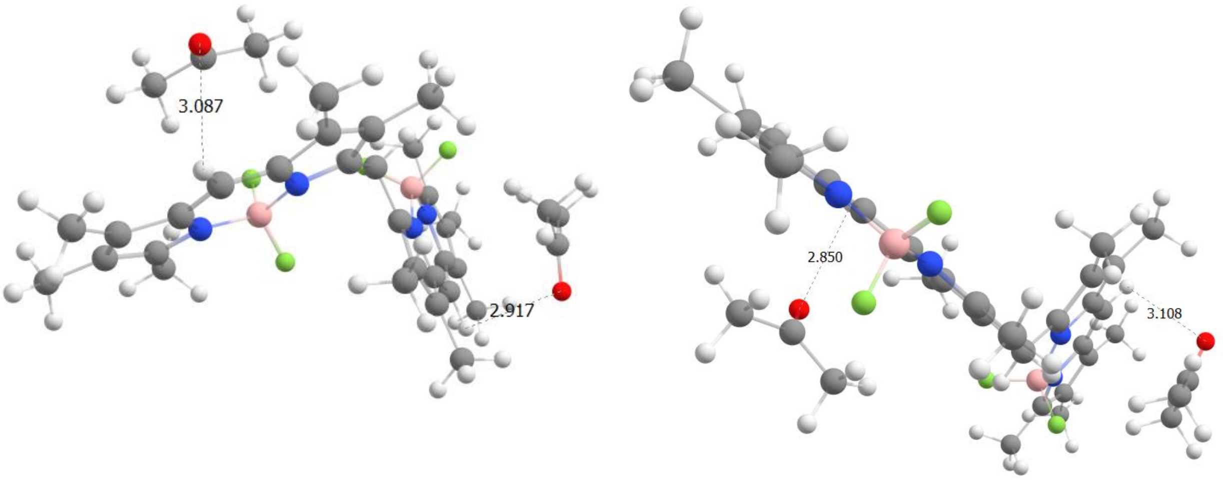

a *b*

*CH_2-bis(BODIPY)**1**·2acetone* *CH_2-bis(BODIPY)**2**·2acetone*

Figure S10. Optimized CH_2-*bis*(BODIPY)·2acetone structures (**1**-a, **2**-b).

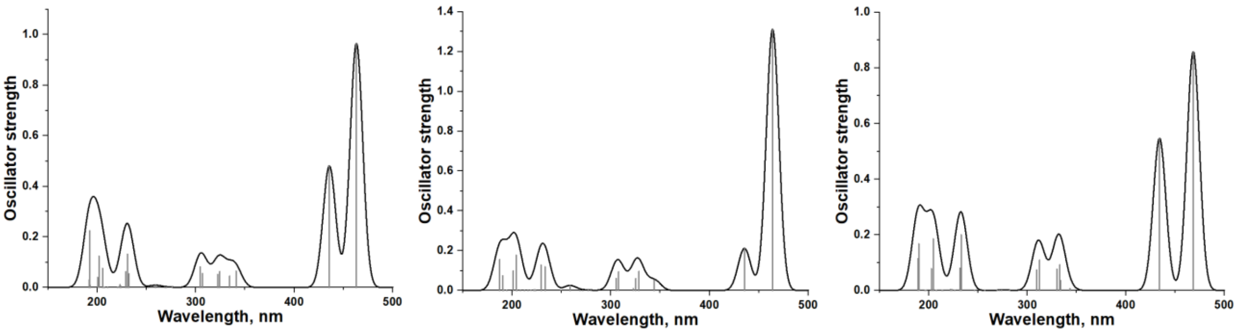

a *b* *c*

Figure S11. TDDFT spectra of CH_2-*bis*(BODIPY)**3**·2acetone (a), CH_2-*bis*(BODIPY)**2**·2acetone (b), and CH_2-*bis*(BODIPY)**1**·2acetone (c).

References

1. Antina, L.A.; Ksenofontov, A.A.; Kalyagin, A.A.; Antina, E.V.; Berezin, M.B.; Khodov, I.A. Luminescent properties of new 2,2-, 2,3- and 3,3-CH2-bis(BODIPY)s dyes: Structural and solvation effects. *Spectrochim. Acta Part A Mol. Biomol. Spectrosc.* **2019**, *218*, 308–319. https://doi.org/10.1016/j.saa.2019.03.117.
2. Riddick, J.A.; Bunger, W.B.; Sakano, T.K. *Organic solvents*: *Physical properties and methods of purification*, 4th ed.; John A. Riddick, William B. Bunger and Theodore K. Sakano, Eds.; Wiley: New York, NY, USA; Chichester, UK, 1986; ISBN 978-0471084679.
3. Haidekker, M.A.; Brady, T.P.; Lichlyter, D.; Theodorakis, E.A. Effects of solvent polarity and solvent viscosity on the fluorescent properties of molecular rotors and related probes. *Bioorganic Chem.* **2005**, *33*, 415–425. https://doi.org/10.1016/j.bioorg.2005.07.005.
4. Antina, L.A.; Kalyagin, A.A.; Ksenofontov, A.A.; Pavelyev, R.S.; Lodochnikova, O.A.; Islamov, D.R.; Antina, E.V.; Berezin, M.B. Effect of polar protic solvents on the photophysical properties of bis(BODIPY) dyes. *J. Mol. Liq.* **2021**, *337*, 116416. https://doi.org/10.1016/j.molliq.2021.116416.
